# Codeveloping a Clinical Pathway for Recurrent Clostridioides difficile Infection With Primary Care Providers: User-Centered Design Study

**DOI:** 10.2196/93032

**Published:** 2026-09-30

**Authors:** Ritika Kamlesh Patel, Rose Franz, Jordan Tate, Jennifer Coppens, Matthew Do Rego, Christopher Smith, Lauretta Ovadje, Karen Wong, Lynora Saxinger, Dina Kao

**Affiliations:** 1 Division of Gastroenterology, Department of Medicine and Dentistry, University of Alberta, 2-24 Zeidler Ledcor Center, 8540-112 St NW, Edmonton, AB, T6G 2P8, Canada, 1 780-248-1342, 1 780-642-4163; 2 Physician Learning Program, Faculty of Medicine and Dentistry, University of Alberta, Edmonton, AB, Canada; 3 Department of Family Medicine, Faculty of Medicine and Dentistry, University of Alberta, Edmonton, AB, Canada; 4 Health Systems Knowledge and Evaluation, Acute Care Alberta, Edmonton, AB, Canada

**Keywords:** Clostridioides difficile infection, clinical pathway, codevelopment, primary care providers, fecal microbiota transplant

## Abstract

**Background:**

*Clostridioides difficile* infection (CDI) is the most common cause of health care–associated infectious diarrhea; however, variability in laboratory testing results in uncertainty in diagnosis and clinical decision-making. Recurrent CDI (rCDI), defined as the return of infection following initial treatment, continues to present significant clinical challenges due to limited therapeutic options. Although health care providers are familiar with vancomycin as first-line therapy for CDI, many are less familiar with fecal microbiota transplantation (FMT), despite its recommendation in clinical guidelines for rCDI management. Consequently, primary care providers (PCPs) may experience uncertainty regarding diagnostic test interpretation, treatment selection, and referral for FMT, highlighting the need for practical clinical tools to support evidence-based management.

**Objective:**

This study will support the standardization of care and improve patient treatment experiences in Alberta by identifying barriers and facilitators encountered by PCPs in managing CDI and rCDI, using these findings to codevelop a provincial rCDI clinical pathway.

**Methods:**

This qualitative study investigated the codevelopment process of a provincial rCDI clinical pathway between the study team and intended end users. A draft pathway was developed by the study team comprising gastroenterology and infectious disease specialists, followed by an iterative end user–centered design approach. PCP participants completed 2 rounds of 60‐ to 90-minute virtual, one-on-one, semistructured interviews incorporating scenario-based usability testing with standardized clinical cases. Using a think-aloud approach, participants evaluated the pathway’s clarity, diagnostic decision-making, treatment recommendations, referral processes, and integration into clinical workflows. Feedback was analyzed using thematic analysis, informed iterative revisions were made to the pathway and accompanying user guide, and they were reevaluated in a second round of interviews.

**Results:**

Out of 30 invited PCPs, 3 (10%) participated in this study. During the first round of interviews, participants identified challenges interpreting PCR-positive vs toxin-positive test results, navigating the pathway layout, locating embedded hyperlinks, and understanding FMT referral processes. Iterative, feedback-driven revisions addressed these barriers by improving diagnostic guidance, simplifying navigation, increasing hyperlink visibility, and clarifying referral timing with a user guide. During the second round of interviews, all participants reported that modifications improved CDI test result interpretation, streamlined navigation, enhanced workflow integration, and increased confidence in applying the pathway to clinical scenarios. The final version of this pathway was well received for its clarity, readability, and clinical utility, and was viewed as a valuable educational resource for supporting evidence-based management of CDI.

**Conclusions:**

This study demonstrated the feasibility and value of codeveloping a provincial clinical pathway with physician partners for managing CDI and rCDI. The resulting tool was clear, practical, and aligned with primary care workflows. This iterative, user-centered design was useful for developing clinically relevant decision support tools. Future research should evaluate the pathway’s implementation and impact on physician practice, patient care, and clinical outcomes using quantitative and qualitative methods.

## Introduction

*Clostridioides difficile* infection (CDI) is a significant health care priority, representing the most common causes of health care–associated infectious diarrhea, commonly triggered by antibiotic use [1]. Gaps in knowledge around CDI diagnosis and recurrent CDI (rCDI) management have contributed to suboptimal and delayed treatments [2,3]. Specifically, CDI is diagnosed in patients who have diarrhea and a positive stool test for toxigenic *Clostridioides difficile*. Diagnostic confusion arises because there are two main categories of laboratory assays: (1) an enzyme immunoassay for toxins A and/or B with high specificity but low sensitivity (33%‐65%) [4], and (2) a polymerase chain reaction (PCR)–based assay with higher sensitivity (approximately 90%) but much lower specificity, as it cannot differentiate between active infection and asymptomatic colonization [5]. While most practitioners are comfortable with vancomycin as the first-line therapy for CDI, most are not familiar with guideline-recommended fecal microbiota transplantation (FMT) for patients with at least 2 recurrences [6,7]. Not only is FMT an efficacious treatment option, it is also the most cost-effective strategy for treating rCDI [8]. In a retrospective study in Alberta, timely FMT, defined as administration after the second recurrence, was associated with mean per-patient cost savings of approximately CAD $29,842 (CAD $1=US $0.71 as of September 23, 2026) compared with delayed FMT [9]. Additional challenges exist as many primary care providers (PCPs) are unsure when to refer [10] or how to access FMT.

Clinical pathways are evidence-based tools designed to translate clinical practice guideline recommendations into routine practice while standardizing and coordinating patient care within a multidisciplinary health care setting [11]. A study evaluating the implementation of a clinical pathway for liver surgery found that its use significantly reduced hospital stays and postoperative costs without increasing postoperative morbidity [12]. However, traditional top-down dissemination of clinical practice guidelines often fails to address potential barriers to their development, dissemination, and implementation [13,14]. As such, it is crucial to involve end users in codeveloping this rCDI pathway to optimize guideline implementation and timely care for rCDI patients.

## Methods

### Study Overview

This study describes the process of cocreating a provincial clinical pathway to support physicians in the management of patients with CDI (including rCDI) in Alberta, Canada, while highlighting key aspects of CDI care, including diagnosis, treatment, and referral processes.

### Ethical Considerations

Ethics approval was obtained from the University of Alberta Research Ethics Board (Pro00130489). All participants signed a written consent form and agreed to be video recorded during the interviews.

### Physician Recruitment

A purposive sampling strategy was applied to recruit participants. PCPs were identified with the support of the Alberta Health Services (AHS) Primary Health Care Integration Network, interested primary care networks, the Alberta Medical Association, and the academic Department of Family Medicine at the University of Alberta. From this pool, PCPs with experience referring rCDI patients were contacted via email to assess their interest in participating. The FMT nurse navigator distributed consent forms and coordinated interviews.

### Study Design and Codevelopment Process

We used a qualitative methodology to gather empirical data on the optimal format and clinical content of the proposed pathway. The draft provincial rCDI clinical pathway was first developed by the study team consisting of specialists in gastroenterology and infectious diseases in collaboration with the University of Alberta’s Physician Learning Program. To facilitate use in clinical practice, the pathway incorporated direct links to existing provincial and Canadian clinical resources, including the Alberta Referral Directory, Bugs & Drugs [15], and other relevant guidance documents, allowing clinicians to access additional information as needed. This draft served as a low-fidelity prototype intended for iterative testing and refinement according to feedback from the end users. A user-centered design approach was also taken, as scenario-based usability testing and iterative prototyping can reveal cognitive and workflow mismatches that are not readily apparent through guideline development alone. Codevelopment activities consisted of 2 rounds of scenario-based usability testing conducted through one-on-one virtual 60‐ to 90-minute interview sessions with PCPs. The sessions followed a semistructured format, framed by an interview guide with case scenarios in addition to open-ended questions. A separate interview guide was designed for each respective interview.

Prior to the first interview, PCPs were provided with the initial draft of the provincial rCDI pathway. During the interview, participants were asked to apply the draft pathway to 2 standardized patient case scenarios representing common but clinically challenging rCDI presentations. Using a think-aloud approach, PCPs described how they interpreted pathway steps, made treatment decisions, and determined referral appropriateness while navigating the tool.

The interviewer probed for clarity, decision confidence, points of hesitation, perceived ambiguities, and workflow misalignments. Particular attention was paid to how physicians interpreted diagnostic test results (PCR vs toxin), distinguished recurrent cases from new infections, and identified when to reassess for alternative causes of diarrhea.

Following the first interview, usability findings were synthesized into structured summary tables and task success matrices capturing decision points, areas of confusion, drop-off locations within the pathway, and suggested revisions. These artifacts were used to guide iterative pathway redesign, including modifications to terminology, visual hierarchy, navigation cues, hyperlink visibility, and referral timing.

The revised pathway and an accompanying user guide were then retested with the same end user PCPs in a second round of interviews to assess whether prior usability issues had been resolved and to validate improvements in clarity, workflow integration, and clinical feasibility.

### Data Collection and Analysis

The AHS Health System Knowledge and Evaluation team, acting as additional independent qualitative analysts, collaborated with the research team to design interview guides including sample case scenarios, facilitate interviews, and conduct data analysis. Verbatim transcripts generated from Microsoft Teams recordings were analyzed using a thematic analysis framework. To ensure methodological rigor, coding was conducted independently by a member of the study team and a member of the external AHS Health System Knowledge and Evaluation team. Discrepancies in code applications were resolved through consensus and consultation with the broader study team rather than using formal quantitative interrater reliability measures.

Theme derivation followed an inductive approach, progressing from initial open coding to group codes into broader, descriptive categories representing specific workflow barriers or design challenges. These categories were then structured into overarching thematic maps (Figures S1 and S2 in Multimedia Appendix 1) to visually map the relationships between diagnostic interpretation, pathway navigation, clinical workflow, and implementation feasibility.

In addition to thematic qualitative analysis, usability data were analyzed using task-based evaluation methods commonly used in human-centered design. Task success and failure were documented alongside qualitative observations and participant quotations. This dual analytic approach enabled triangulation between what physicians reported, how they navigated the pathway, and where design-related barriers emerged. Findings were iteratively mapped back to pathway revisions to ensure that identified usability issues directly informed design changes rather than remaining descriptive only.

## Results

### Participants

Of the 30 PCPs invited, 3 provided consent and subsequently participated in one-on-one interviews. Participant gender and practice history are presented in Table 1.

**Table 1.** Participant characteristics (N=3).

Characteristic	Participants, n (%)
Male	2 (66.7)
Health care specialty and practice setting	
Primary care	3 (100)
Plus emergency medicine	1 (33.3)
Plus geriatrics	1 (33.3)
Urban	2 (66.7)
CDI^a^ patient volume (per year)	
**<**10 patients	2 (66.7)
**>**10 patients	1 (33.3)

a CDI*: Clostridioides difficile* infection.

### Interview Sessions

#### PCP Interviews Round 1

Three PCPs participated in 2 rounds of semistructured, one-on-one interviews. All 3 participants worked in primary care settings with annual patient volumes exceeding 500.

The first set of interviews were conducted between May 29, 2024, and June 6, 2024. The initial draft of the pathway incorporated direct access links to several resources: (1) Bugs & Drugs, an evidence-based antimicrobial reference guide for health care professionals in Alberta [15]; (2) the AHS diarrhea pathway, a clinical guide for managing diarrhea in primary care, including a step-by-step approach with investigations and management; and (3) patient education resources. A warning sign–flagged hyperlink was incorporated to distinguish fulminant CDI from severe nonfulminant CDI and to direct clinicians to the inpatient CDI management pathway for cases requiring urgent care. The pathway begins by outlining clinical criteria that define “suspected new or recurrent CDI,” followed by a recommendation to obtain a *C difficile* diagnostic test in the appropriate context. Subsequent management is guided by the test results, which may be (1) toxin positive, (2) PCR positive with additional risk assessment, or (3) both toxin and PCR negative. Based on these findings, the pathway specifies what treatment is required and outlines the recommended clinical actions for CDI recurrence, including management for first, second, and third episodes. The pathway explicitly indicates FMT referral at the third episode (or second recurrence). Further recommendations are based on treatment response.

The initial and final versions of the pathway can be found in Figures 1 and 2, respectively. Visually, each major phase of decision-making is separated by dotted lines to delineate distinct stages of progression. Individual outcomes are presented in separate boxes to emphasize branching pathways. Embedded hyperlinks within the outcome boxes direct users to supplementary informational resources when required.

**Figure 1.**
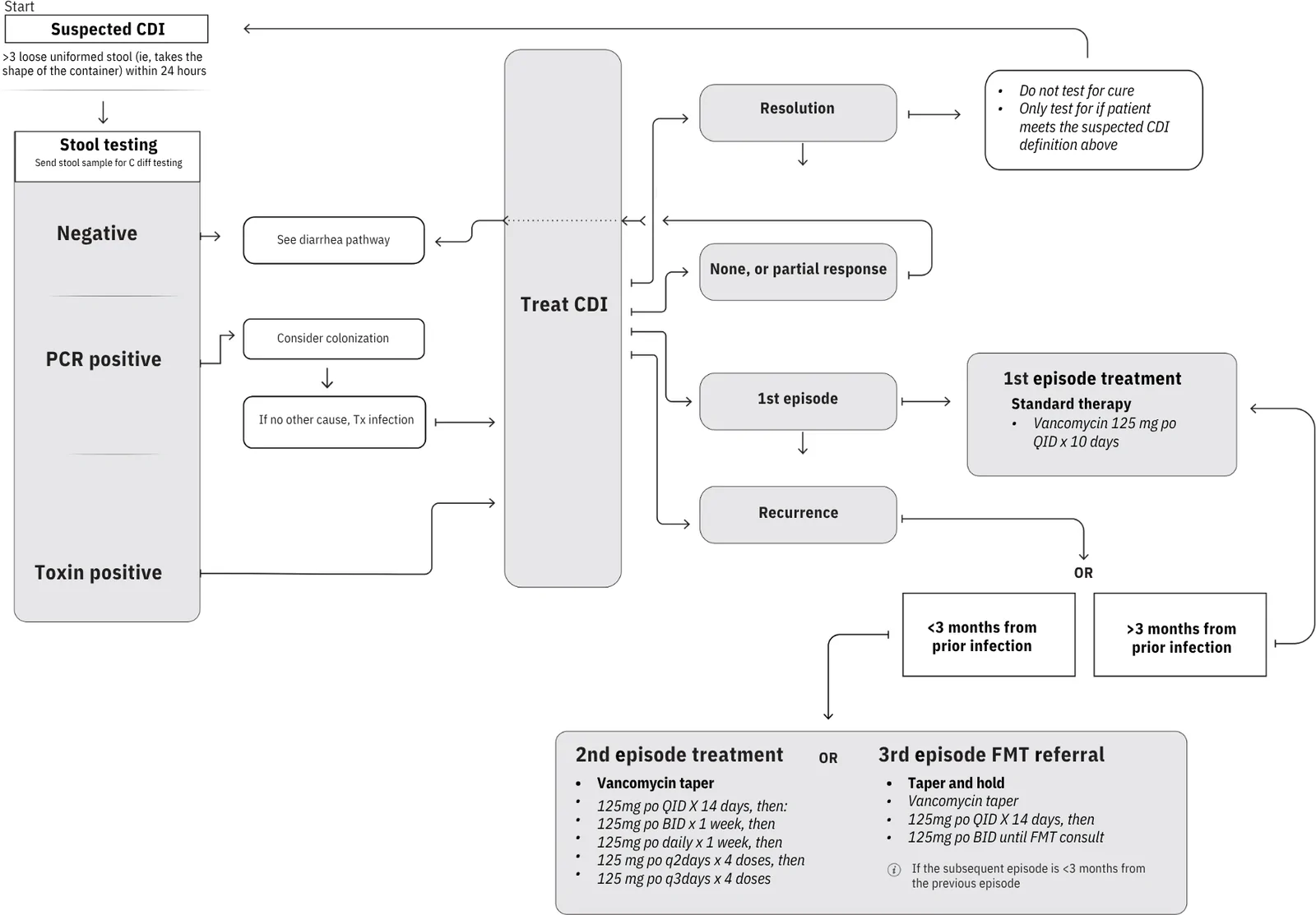
Initial draft of the recurrent *Clostridiodes difficile* infection (rCDI) clinical pathway prior to finalization. BID: twice a day (*bis in die*); C diff: *Clostridiodes difficile*; CDI: *Clostridiodes difficile* infection; FMT: fecal microbiota transplantation; PCR: polymerase chain reaction; po: by mouth (*per os*); q2days: every 2 days (*quaque* 2 days); q3days: every 3 days (*quaque* 3 days); QID: 4 times a day (*quater in die*); Tx: treatment.

**Figure 2.**
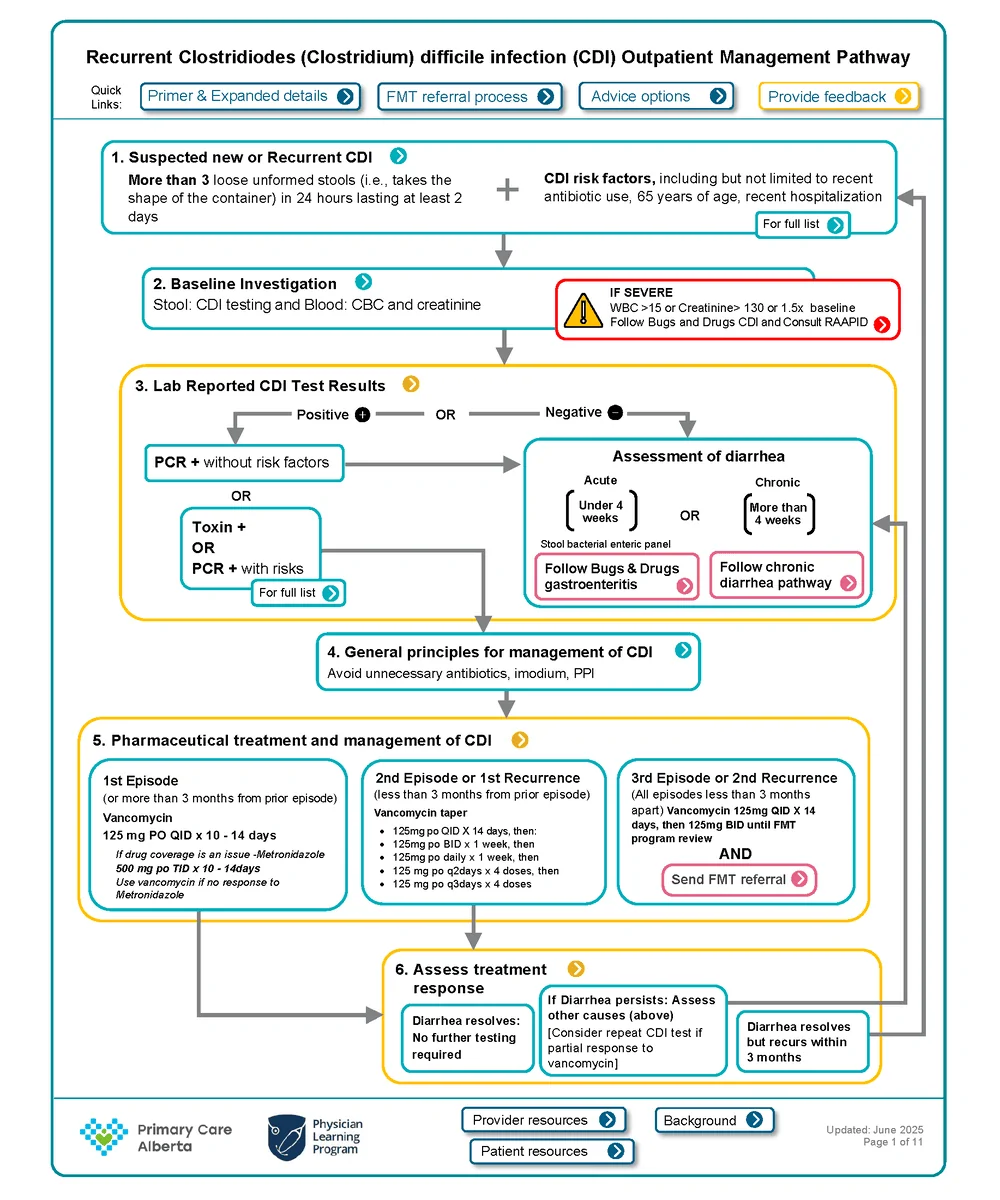
Final version of the recurrent *Clostridiodes difficile* infection (rCDI) clinical pathway. BID: twice a day (*bis in die*); CBC: complete blood count; FMT: fecal microbiota transplantation; PCR: polymerase chain reaction; po: by mouth (*per os*); PPI: proton pump inhibitor; q2days: every 2 days (*quaque* 2 days); q3days: every 3 days (*quaque* 3 days); QID: 4 times a day (*quater in die*); RAAPID: referral, access, advice, placement, information, destination; WBC: white blood cell.

The first interview focused on eliciting participant feedback on the clarity and usability of the pathway. Participants were asked to apply the pathway to 2 patient scenarios and describe how they would manage each case, including the need for FMT referral and the appropriate timing and process for referral.

Task-based usability outcomes from the scenario-based interviews are summarized in Table 2. Participants completed a series of predefined clinical decision-making tasks, including distinguishing between new and recurrent CDI, interpreting diagnostic test results, selecting the appropriate management pathway, identifying the appropriate timing for FMT referral, and navigating the pathway to relevant supporting resources. Areas of task difficulty identified during the first round of usability testing informed iterative revisions to the pathway prior to the second round of interviews.

**Table 2.** Participant performance on predefined usability tasks during pathway evaluation (N=3).

Usability task	Round 1	Round 2
Accurate classification of recurrence vs new infection	2/3 success; 1/3 partial success^a^	3/3 success
Interpretation of PCR^b^-positive vs toxin-positive results	0/3 success; 3/3 partial success^a^	3/3 success
Appropriate treatment selection	3/3 success	3/3 success
Correct identification of FMT^c^ referral thresholds	2/3 success; 1/3 partial success^a^	3/3 success
Confidence^d^ in next-step decision-making	2/3 success; 1/3 partial success^a^	3/3 success

a Partial success means the participant ultimately reached an appropriate decision but required clarification, showed significant hesitation, or completed only 1 of the 2 scenarios correctly.

b PCR: polymerase chain reaction.

c FMT: fecal microbiota transplantation.

d Confidence was not measured using a numerical scale during the interviews. This row is based on retrospective coding of participant hesitation, requests for clarification, and ability to independently identify the appropriate next step.

Complementary qualitative findings are presented in Figure S1 in Multimedia Appendix 1, which illustrates the thematic map generated from the first round of interviews. The thematic analysis identified barriers related to pathway design, structural organization, diagnostic test interpretation, navigation, and referral processes. Together, the usability and qualitative findings informed iterative refinements to improve the pathway’s clarity, usability, and alignment with practitioner workflows.

*I guess one of my questions would be about the testing. I think there needs to be something on this path...that talks about the difference between toxin testing and PCR testing, as I suspect I am not alone as a family physician in wondering when each of those happens*.

[Participant 3]

*That is a little bit ambiguous in terms of the PCR testing, that I would...clean that up, but just so that it makes it a little bit more clear cut that that’s what you mean...I understand now that it’s explained, but I wouldn’t have thought that straight away*.

[Participant 2]

Participants reported difficulty navigating the draft pathway, as the dotted lines gave the impression that sections were separate and did not allow users to circle back through the algorithm. Consequently, PCPs were uncertain about when to assess patients for alternative causes of diarrhea (Figure 1).

*I’m wondering if...there’s a way to link back from...this algorithm...it kind of makes it look like once you’re in the “treat C diff” you don’t go back, although I guess down at the bottom under treatment outcome you go down to “Assess other causes”*.

[Participant 1]

PCPs recommended clearer visual cues for hyperlinks, such as the use of consistent icons to indicate their presence. While the draft pathway clearly indicated when patients should be referred for FMT, 2 PCPs noted concerns about the referral process itself. Additionally, the placement of the FMT referral button did not clearly convey if the referral should occur concurrently with vancomycin treatment.

*When I kind of first looked at the pathway it just kind of seemed a little bit out of place. Like I didn’t know what, like where that kind of related, if you don’t click the links*.

[Participant 1]

*Just in case there is anyone that perceives that as an ambiguity...If you send the FMT referral, if you put that as the first thing, and then you do that concurrently*.

[Participant 2]

All participating PCPs reported that the pathway enabled them to distinguish between a new CDI infection and a recurrent infection. They also indicated that the pathway clearly outlined the treatment approach for both scenarios. Participants recommended including an appendix or user guide containing definitions and additional information to avoid having to consult an external source.

*So on a lot of the specialist link pathways and provincial pathways and stuff they have the page like this, and then there’s a link within here that just leads to almost an appendix that it details out risk factors or details out like higher risk features in more detail kind of things so that I don’t have to go to a second source*.

[Participant 1]

PCPs were asked whether their treatment approach differed when a patient’s results were PCR positive vs toxin positive, and all indicated that it did. PCPs considered a PCR-positive result in patients without risk factors indicative of colonization and did not initiate CDI treatment. In cases where patients showed no response or a partial response to CDI therapy, PCPs reported extending treatment duration, conducting additional testing for alternative causes, and referring the patient as appropriate.

*I would do more of a work up...broaden my differential and look at things again. If they were toxin-positive and didn’t respond...I know the toxin takes a while to go away, even with treatment. So it would kind of depend how long, I might consider retesting for C diff, and...if the toxin was negative, [it] would be more helpful than if it’s positive. But ultimately if I’m just really not sure what to do, I would usually do a connect MD phone consult or something like that*.

[Participant 1]

When asked about the potential use of patient education materials linked to the pathway, all participating PCPs indicated they would use such resources. Additional feedback highlighted variability in clinical workflows, including differences between practices using provincially based electronic medical records or paper charts, which may affect how this clinical pathway is accessed. Some PCPs noted that certain pathway content was not fully aligned with information in Bugs & Drugs, which highlighted the need for consistency with existing reference materials.

*That could be a very big hindrance in following this pathway [the misalignment with Bugs & Drugs]...I’ve dealt with some really recurrent C diff so I don’t put people on Metronidazole. I put them on Vanco as my rule of thumb...whenever I have students and I teach them this because I’m also a precept at the university. I try and explain this and they said “no, no, but Bugs & Drugs says...” and I said “yeah, that’s fine.” But that’s not what we should do...*

[Participant 2]

Based on participant feedback, we incorporated additional resources, including links to alternative pathways for varying clinical scenarios and detailed information on the FMT referral process. The revised version was then presented to the same physicians during a second interview to further refine the pathway.

#### PCP Interviews Round 2

The second round of interviews was conducted between February 27, 2025, and March 11, 2025. Prior to these interviews, PCPs were provided with the revised provincial rCDI clinical pathway along with the user guide, which had been developed based on feedback from the first round of interviews.

Figure S2 in Multimedia Appendix 1 presents the thematic map derived from analysis of the second interview, focused on evaluating the revised pathway and assessing whether ambiguities were resolved. Overarching themes reflected improved diagnostic clarity, navigation, referral processes, and considerations related to workflow integration and implementation.

Task-based usability testing demonstrated improvements across all predefined tasks following pathway revision (Table 2), supporting the qualitative findings that participants experienced greater clarity in diagnostic interpretation, referral processes, and pathway navigation.

It was much more readable and more consistent with the other pathways.

[Participant 1]

The participating PCPs indicated that the revised pathway had improved clarity in diagnostic test interpretation, differentiation between new and recurrent CDI, referral timing, and overall ease of navigation.

*It [the pathway] does [clarify test results]. Just the addition of the two categories on the third step is useful*.

[Participant 3]

*I think it makes it more clear about what’s a recurrence versus what’s a new infection...Yeah, I thought that section was definitely clarified. For sure, and easier to read*.

[Participant 1]

One participant suggested removing the “If severe” box because the guidance provided in Bugs & Drugs was consistent with the information already in the pathway.

*Because presumably, if your white blood cells are high and your creatinine’s high, you also need the positive CDI test...if it’s C. diff, then you go down to five. And it doesn’t change what you do for that first episode, apart from the clinical assessment of maybe they need hydration or admission or something like that...but the instructions to follow Bugs & Drugs CDI, you’re kind of doing that anyway. Going through the pathway, so you don’t need to shunt someone else to a different source when it’s exactly the same as what’s later on in the pathway*.

[Participant 1]

The user guide (Multimedia Appendix 2) was perceived as a comprehensive supplement to the pathway and was particularly helpful in diagnostic test interpretation, including the distinction between PCR-positive and toxin-positive results. However, participants indicated that the user guide added little additional value for CDI management or differentiating new vs recurrent infections, as the pathway itself already provided sufficient clarity.

*That’s [the User Guide] exactly what provides the clarity for sure*.

[Participant 2]

*Truthfully, it’s pretty well laid out in the pathway. So really, in terms of the treatment itself, I don’t know that the User Guide contributes a lot to the treatment component of it specifically...given that the pathway’s quite clear on what the treatment would and should be*.

[Participant 3]

This sample of PCPs viewed the pathway as a valuable educational resource and recommended several refinements to the accompanying user guide, including guidance for managing patients with a vancomycin allergy and providing contact information for physicians requiring further support. A recurring theme across interviews was how and when the pathway would be used. One suggestion was to attach or link the pathway to a positive CDI laboratory result, ensuring that physicians have the provincial rCDI clinical pathway and user guide available at the point of care.

*That might be something to put on the PCR results of C diff testing at the bottom. Say, “please see Provincial Pathway for CDI, for recurrent CDI management.” Because a lot of times someone comes in with diarrhea, you know, I think a lot of people are like, OK, C diff, I’m going to check for it and then they get the results back and they’re like, “what do I do with this now?” And if you have the link directly there in the results...they’re much more likely to look at it*.

[Participant 1]

*It’s a good teaching tool in that sense, to physicians as well, because that will be implicit if someone follows the pathway and follows that course...it helps teach for the future*.

[Participant 3]

Given the small sample size, thematic saturation may have been limited. However, recurring themes were consistently identified across interviews, supporting the credibility of the findings within this exploratory sample.

## Discussion

### Principal Result

Physician partners in our study identified important gaps in the current CDI clinical workflow, including inconsistencies in referral patterns and challenges in accessing consistent information. Through an iterative co-creation process with PCPs, we developed a physician-centered pathway to guide diagnosis, management, and referral for rCDI. The resulting pathway was perceived by participants as clinically relevant, practical, and easy to use, highlighting the feasibility of using a co-creation framework to design practical, end user–aligned clinical pathways. However, our participating physicians were all highly engaged, academically affiliated, and had prior experience referring rCDI patients, and results may differ if we had included participants in rural practices or those without prior experience with rCDI patients. Nevertheless, as a regional, qualitative development study, our results demonstrate the value of the co-design process. Future studies should evaluate pathway uptake and impact on clinical practice, referral patterns, health care utilization, and patient outcomes.

### Current Landscape

Prior to this project, no provincial clinical pathway existed for CDI management in Alberta. While CDI practice guidelines have been published by professional societies, access to FMT in Canada remains limited, and most practitioners do not know when or how to access this treatment [16]. Clinical pathways may address these gaps by translating evidence into practice and improving access to emerging treatments. Integration of pathway content into electronic health records has been proposed as a low-effort strategy to enhance awareness [17], which informed the development of our clinical pathway.

### Co-Creation Process

Co-creation is gaining recognition as a valuable way to bring together stakeholders to promote collaboration, support knowledge dissemination, and exchange perspectives [18]. In practice, coordinating multiple health care professionals during pathway development can be challenging. The complexity of the clinical problem, intervention, and implementation, including the need to align pathway goals across members of the health care team and ensure stakeholder engagement, may either facilitate or hinder both pathway development and implementation [19]. Also, a lack of leadership and limited intrinsic motivation can be barriers in a clinical setting [19]. Therefore, involving a specialist in a relevant health care field is essential for providing guidance and structure in the initial development of care pathways [20]. Across specialties, pathways have demonstrated effectiveness in standardizing patient care, improving timeliness, and reducing costs while not negatively affecting overall patient health outcomes [12,21]. For effective knowledge mobilization, particularly between specialists and PCPs, codevelopment provides an important mechanism for generating tools that are relevant and acceptable to stakeholders, more usable, and more likely to be successfully implemented within health care settings [22,23].

Embedding human-centered design methods allowed us to translate physician feedback into iterative refinements that improved our pathway’s clarity and usability. Although the initial draft was developed by the multidisciplinary research team using existing evidence and specialist expertise, repeated engagement with PCPs allowed their perspectives to inform iterative pathway refinement. Future work could involve PCPs earlier in pathway conceptualization, enabling them to shape pathway content from the outset while further strengthening stakeholder ownership and supporting implementation.

### Clinical Pathway Efficacy

The existing literature examining the co-creation of clinical pathways with PCPs is limited. However, studies evaluating clinical pathways more broadly demonstrate that their implementation can yield numerous positive outcomes across the journey of care, from diagnosis to treatment. One study assessing the impact of a clinical pathway reported a marked reduction in health care–associated CDI rates following the introduction of a mandatory pathway [24]. The investigators attributed this reduction to decreased rates of misdiagnosis, reducing the use of resources and health care costs and promoting the delivery of appropriate patient care.

Evidence from other studies further emphasizes the utility of clinical pathways in other areas. A study conducted in the United States found that implementing clinical pathways for nutritional management and lower airway inflammation in patients with cystic fibrosis resulted in more standardized and uniform care [25]. Additionally, a systematic review from Europe reported that care pathways enhanced health care staff knowledge, improved interprofessional documentation, and strengthened both communication and team relationships [26]. Across these studies, a recurring theme was the necessity of a multidisciplinary approach in developing and implementing care pathways, which can effectively support collaboration within interprofessional teams and enhance the quality of patient care [25,26].

### Limitations

A key limitation in this study is the low number of participants. Potential participants may have been discouraged by the required time commitment of up to 4 hours, which can be a significant challenge for a busy clinician. Selection bias may also have been introduced in this exploratory codevelopment study; as participants were recruited through professional and academic networks, all 3 PCPs were academically affiliated and had prior experience treating and referring rCDI patients. Self-selection bias was also a concern, as those who agreed to participate may have held stronger opinions regarding clinical pathways or a greater interest in quality improvement initiatives. Although this small sample size was appropriate for iterative pathway development, the findings may not fully reflect the perspectives of physicians practicing in other settings, those who have not managed rCDI patients, or other potential end users such as nurses, pharmacists, or patients. A greater diversity of participants may have provided valuable perspectives for further refining this pathway and its implementation. A higher number of participants would also permit specific feedback to be quantified to substantiate our qualitative analyses. As such, the findings should be viewed as exploratory and may not be transferable to community-based, rural-based, or less academically engaged practitioners and allied health care team members.

Supporting PCP engagement in research is essential to developing systemic interventions that mitigate clinical burdens and foster robust, interdisciplinary support networks across medical specialties [27,28]. Enhancing future PCP research participation requires a multipronged approach, which may include equitable financial compensation, reduced interference with daily practice routines, and enhanced institutional backing [29,30]. Future pathway refinement should also incorporate perspectives from additional stakeholders, including nurses, pharmacists, and a more diverse group of physicians, to strengthen the pathway implementation and transferability.

Methodological limitations should also be noted. The interview guides were not pilot tested, potentially limiting our ability to assess and refine the validity of their content [31]. Interviews are a valuable method for gathering information; however, the quality of data obtained through interviews can vary considerably depending on the researcher’s qualifications, skills, and familiarity with the research topic.

### Conclusions

The co-creation of clinical pathways with physician partners represents a feasible approach for developing clinically relevant decision support tools for the management of rCDI. Future research should explore the acceptability and effectiveness of alternative formats for the pathway to ensure these resources remain accessible and relevant across diverse primary care settings. As the rCDI clinical pathway is introduced into the provincial referral directory, prospective evaluation will be critical to assess its impact on clinical decision-making, referral practices, implementation, and patient outcomes, while informing future refinement of the pathway.

## Supplementary material

10.2196/93032 Multimedia Appendix 1 Supplementary figures 1 and 2 of physician interview thematic maps.

10.2196/93032 Multimedia Appendix 2 Pathway user guide.

## Abbreviations

AHS: Alberta Health Services
CDI: *Clostridioides difficile* infection
FMT: fecal microbiota transplantation
PCP: primary care provider
PCR: polymerase chain reaction
rCDI: recurrent *Clostridioides difficile* infection
