# Supplementary material for: Codeveloping a Clinical Pathway for Recurrent Clostridioides difficile Infection With Primary Care Providers: User-Centered Design Study
Source: Interact J Med Res. 2026 Sep 30;15:e93032. doi: 10.2196/93032 (PMC13626190; doi:10.2196/93032)

Supplementary Figure 1. Thematic map for Physician interview 1.

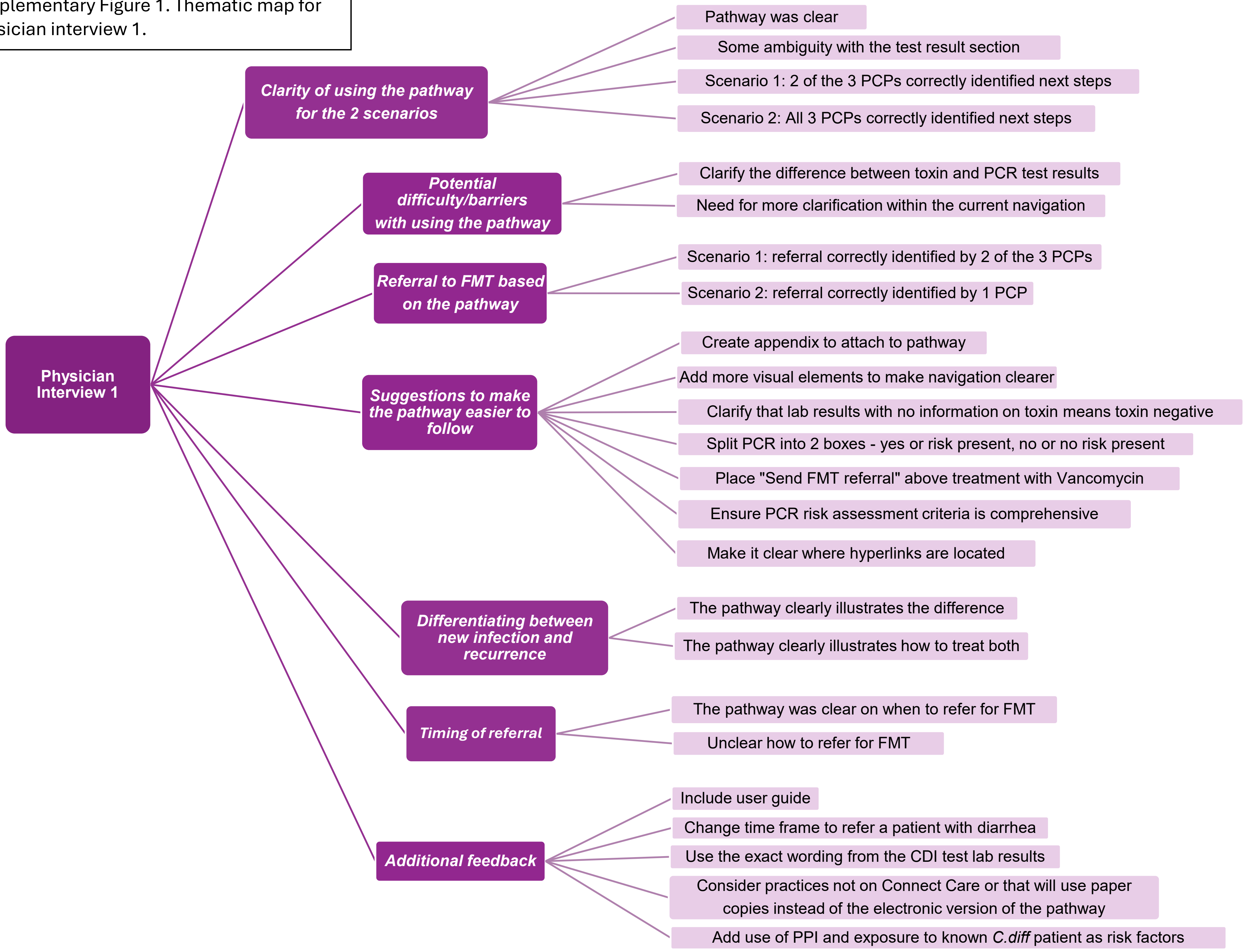

Supplementary Figure 2. Thematic map for Physician interview 2.

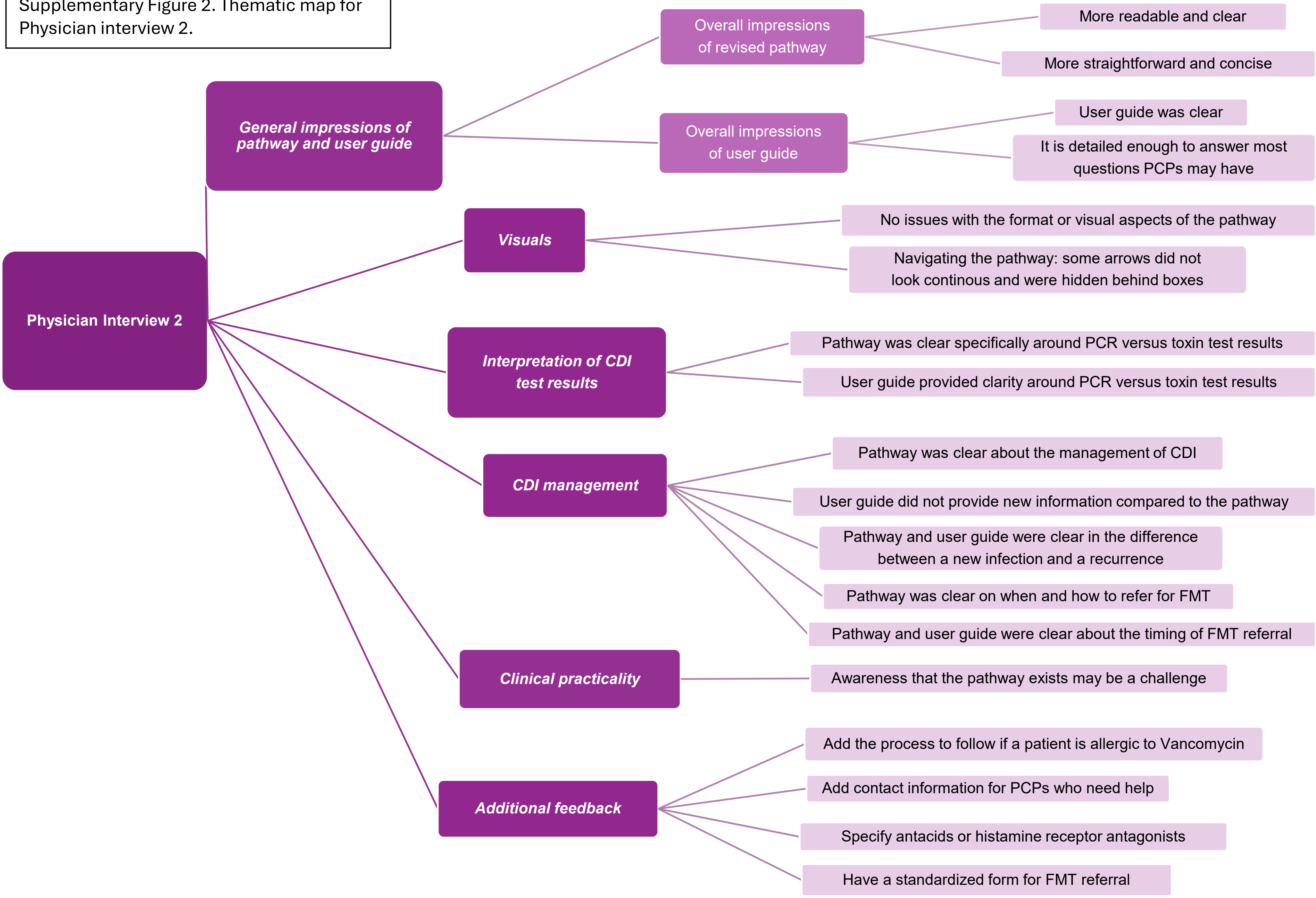

Supplement: Supplementary file 1 [file ijmr-v15-e93032-s001.zip › Physician interview thematic maps 1 + 2.pdf]
