# Supplementary material for: Codeveloping a Clinical Pathway for Recurrent Clostridioides difficile Infection With Primary Care Providers: User-Centered Design Study
Source: Interact J Med Res. 2026 Sep 30;15:e93032. doi: 10.2196/93032 (PMC13626190; doi:10.2196/93032)
Supplement: Supplementary file 2 [file ijmr-v15-e93032-s002.zip › User Guide.pdf]

Recurrent *Clostridioides difficile* infection (rCDI) OUTPATIENT MANAGEMENT PATHWAY PRIMER

This pathway is intended to guide best practice in treating patients with rCDI to ensure that the care is standardized across Alberta and when to consider referral for fecal microbiota transplantation.

- CDI: defined by a positive *C. difficile* test (toxin or PCR) with diarrhea <u>and</u> resolution of diarrhea with anti-CDI treatment.
  - Diarrhea is defined as $\geq$ 3 loose or watery stools/ day (Type 6-7 on the Bristol Stool Chart) within 24 hours persisting for $\geq$2 days.
- *C. difficile* recurrences: defined by episodes separated by < 3 months apart after the completion of anti-CDI treatment for previous episode. An episode which occurs more than 3 months following the completion of anti-CDI treatment for the previous one is, considered a new infection, not a recurrence.
- Most patients with CDI have a history of recent antibiotic use. CDI infrequently occurs in patients without an antibiotic trigger, but this is usually with other risk factors linked to intestinal dysbiosis, such as inflammatory bowel disease, immunosuppression, advanced age, etc. (see complete list of risk factors below).
- Difficulty exists distinguishing between an infection and colonization in those with PCR+ test results
  - If CDI directed treatment is prescribed, it is important to assess the response to anti-CDI treatment. Should there be no or partial response be sure to assess for alternative causes of diarrhea.
- Fecal microbiota transplant (FMT) is considered an investigational therapy by Health Canada. **The only approved indication for FMT is in those with rCDI.**

	**Checklist to guide prior to sending FMT Referral**
□	Confirm patient has 2nd CDI recurrence (see algorithm Box 5).
□	Confirm patient has response to anti-CDI therapy.
□	Confirm patient is not currently on, or is known to be at risk of further antibiotics within 1 month of referral.
□	Send FMT referral. Follow process as indicated below.
□	Ensure patient is maintained on adequate vancomycin suppression after submitting referral (see algorithm Box 4).

EXPANDED DETAILS

1. Suspected CDI or Recurrent CDI

Is defined as: >3 loose uniformed stool (i.e., takes the shape of the container) within 24 hours lasting > 2 days [3]

AND

One or more of the following risk factors:

- Recent Antibiotic Use
- ≥65 year of age
- Recent hospitalization
- Acid Suppression therapy
- Hematologic malignancy
- Neutropenia
- Inflammatory bowel disease
- Serum Albumin <30g/L

- CDI infrequently occurs in patients without a recent antibiotic. CDI should only be considered in those without a noted antibiotic trigger if patient has other risk factors linked to intestinal dysbiosis, such as IBD, immunosuppression, advanced age, etc.
- ⚠ **Severe:** If the patient is suspected to have severe or fulminant CDI (WBC > 15,000 cells/mm3 or serum creatinine level >130 or 1.5X above baseline) please refer to:
  - Bugs and Drugs (www.bugsanddrugs.org/24EC0555-2B8D-4F68-B50A-0EF15142FCAA)

    AND
  - Call RAAPID to consult with a GI or ID specialist.

2. Baseline Investigation

Stool: *C. difficile* test

- *C. difficile* testing is NOT indicated in patients with solid/ formed stool. Lab will reject formed stool specimens.
- *C. difficile* testing is NOT indicated after diarrhea resolution or for test of cure.
- Do not repeat testing for *C. difficile* unless diarrhea resolves then recurs. Lab will reject stool specimen if sent within 7 days of previous stool specimen.
- Do not repeat testing while patients are still on CDI treatment.

3. Lab Reported Test Results

The laboratory follows a 2-step algorithm, since these tests individually have different sensitivities and specificities; they complement each other.

The testing algorithms are not the same in South and North Zones. There is ongoing effort provincially to optimize *C. difficile* testing. Additional changes may be seen in the future.

Calgary/ South Zone

- Stool samples are first screened by an immunoassay to detect glutamate dehydrogenase (GDH) specific for *C. difficile*. The sensitivity of this assay is >99%. If this test is negative, *C. difficile* is ruled out. The GDH antigen test can detect both toxigenic and non-toxigenic strains.
- To confirm the presence of a toxigenic *C. difficile* strain, all GDH-positive specimens are tested by real time polymerase chain reaction (PCR) test for rapid detection of toxin B gene sequences (Cepheid GeneXpert® Dx System). Positive specimens by this toxin B PCR are reported as positive. This PCR method is >99% sensitive and >93% specific.

Lab Reporting

Result	Report reads:
**PCR+**	Test for *C. difficile* toxin B gene POSITIVE by polymerase chain reaction (PCR).<br><br>Repeat *C. difficile* testing within 7 days will not be performed.<br><br>This result can be seen in current infection, colonization, or past infection. If the patient has diarrhea, the diagnosis of *C. difficile* infection and decision to treat should take into consideration other causes. Infection, prevention and control precautions are recommended for any patient with diarrhea.<br><br>For inpatients refer to the *Clostridiodes difficile* order sets in Connect Care (EPIC) or Clinical Knowledge Content Management (CKCM) on AHS insite.
**Negative**	Test for *Clostridioides (Clostridium) difficile* NEGATIVE<br><br>Comment: Screened for *C. difficile* by toxin B gene polymerase chain reaction (PCR).

North/ Edmonton/ Central Zone

All samples are first screened by PCR.

If PCR is positive, it is then subjected to the *QuikChek* Complete test, which simultaneously tests for GDH and toxin by enzyme immunoassay (EIA).

- If both GDH and toxin are positive, this means *C. difficile* toxin is detected.
- If GDH is positive but toxin is negative, this means that toxin is not detected in the sample.

Lab Reporting

Result	Report Reads:	Interpretation
**Toxin +**	Test for *C. difficile* toxin POSITIVE by Enzyme Immunoassay<br><br>Test performed with C. DIFF QUIK CHEK COMPLETE (Enzyme Immunoassay)	*C. difficile* toxin detected
**PCR+**	Test for *C. difficile* toxin POSITIVE by polymerase chain reaction (PCR)<br><br>**(PLEASE NOTE: toxin production was not detected)**<br><br>This result can be seen in colonization, past infection, or possible current infections. If the patient has diarrhea, the diagnosis of *C. difficile* infection and decision to treat should take into consideration other causes of diarrhea.	*C. difficile* toxin B gene detected. **Toxin not detected.**
**Negative**	Test for *C. difficile* NEGATIVE<br><br>Screened for *C. difficile* by toxin gene polymerase chain reaction.	*C difficile* toxin B gene not detected

Additional considerations when result is toxin negative.

PCR+

- A PCR positive test cannot distinguish between colonization or infection. Therefore, it is important to assess whether a patient has any of the risk factors for CDI listed above to determine the pretest probability of a positive test.
- If a patient does not have a risk factor listed above, a positive PCR test more likely represents colonization. In that situation, consider investigating for alternative causes of diarrhea. If a decision is made to treat suspected CDI in this situation, it is crucial to assess response to treatment. If there is only partial or no response, there is likely another etiology driving the diarrhea.

Negative

If result is negative, then patients should have investigation for alternative causes of diarrhea:

- Acute diarrhea (< 4 week): look for potential medications that can cause diarrhea and test for other potential bacterial pathogens by doing Stool C & S (see Bugs & Drugs-hyperlink www.bugsanddrugs.org/57663C22-7AFC-4CD7-B34A-C5ADD266D5AD).

- Chronic diarrhea (≥ 4 weeks), please follow chronic diarrhea pathway (hyperlink www.albertahealthservices.ca/assets/about/scn/ahs-scn-dh-pathway-chronic-diarrhea.pdf

4. General Principles for Treatment and Management of CDI

Consider the following for conservative management of CDI

- Avoid over the counter probiotics.
    - Eating a healthy diet, including fiber, has a greater impact on the gut bacteria.
        - See Fiber Facts- www.albertahealthservices.ca/assets/info/nutrition/if-nfs-fibre-facts.pdf
- Avoid the use of Imodium or Lomotil
- Avoid unnecessary antibiotics
- If antibiotic treatment cannot be avoided, a narrow spectrum drug in the lowest possible dose for the shortest duration is recommended.
- If possible, use over the counter medications to control heartburn and acid reflux. Proton pump inhibitors or PPIs (common brands are Tecta and Pantaloc) can be associated with CDI with long term use.

5. Pharmacological Treatment and Management/ Referral of CDI

Use the following escalation of treatments

First Episode (>3 month from prior episode of infection if present):

- Therapy for the first episode: first line therapy is vancomycin 125 mg PO QID x 10 days.
- Metronidazole 500 mg PO TID x 10- 14 days is an acceptable alternative and can be considered if vancomycin is cost prohibitive. If there is no response to metronidazole, switch to vancomycin.

Second Episode/ 1St Recurrence (<3 months from prior episode of infection)

Vancomycin taper

- 125 mg po QID x 14 days, then;
- 125 mg po BID x 1 week, then;
- 125 mg po daily x 1 weeks, then;
- 125 mg po q2days x 4 doses, then;
- 125 mg po q3days x 4 doses

Third Episode/ 2nd Recurrence (All episodes <3 months apart)

- Vancomycin 125 mg PO QID x 14 days then BID x 4 weeks and simultaneously initiate an FMT referral.

  Note: Fidaxomicin could also be considered for treatment of second or third episode. Please be aware that a special authorization (www.ab.bluecross.ca/dbl/pdfs/60015.pdf) will be required for most insurance plans*

FMT Referral:

- FMT is considered an investigational therapy by Health Canada. The only approved indication for FMT is in those with recurrent CDI.
- Recurrences of CDI are defined by episodes separated by <3 months apart. If an episode is separated by >3 months from the previous episode, it is considered a new infection, not a recurrence.
- Current treatment guidelines recommend FMT after the 2nd recurrence, or the 3rd episode of CDI.

Things to consider before FMT referral:

Patient with underlying IBD:

If a patient has only partial response (ie improvement in diarrhea but not complete resolution), then endoscopic evaluation is required prior to FMT referral. This is to help specialists determine if there is active IBD, which may be driving symptoms, as IBD therapy may need to be escalated. As such, these patients should ideally be evaluated by their IBD specialist first.

FMT is used to prevent future recurrence. Therefore, diarrhea should resolve prior to receiving FMT.

- While waiting for assessment by the FMT program, patients should remain on anti-CDI suppression therapy eg. vancomycin 125 mg PO daily or bid to prevent further recurrence after completing 125 mg PO qid dosing for 14 days.
- FMT should be delayed in patients known or suspected to need further antibiotic until the anticipated antibiotic therapy is complete. During this time, they should remain on anti-CDI suppression therapy.
- FMT donor screening and product manufacturing is a complex and costly process and as such FMT treatment should only be given when it has the highest chance of success.

FMT Referral Process:

Submitting FMT referrals is different depending on zone.

<u>On Connect Care</u>

Calgary Clinic:

Enter new order: Ambulatory Referral to Infectious Disease/Gastroenterology

- Class: Internal Referral
- Department Specialty: Infectious disease
- To Department: CGY FMT Microbial Therapy
- To Provider: Dr. Humberto Jijon, Dr. T. Louie

Edmonton Clinic:

Enter new order: Ambulatory Referral to Gastroenterology

- Class: Internal Referral
- Department Specialty: Gastroenterology
- To Department: EDM UAH ZLC FMT
- To Provider: Dr. Karen Wong or Dr. Dina Kao

<u>If NOT on Connect Care:</u>

Calgary Clinic:

Send referral to The Microbial Therapy Clinic

- Fax: 403 355-9751

Edmonton Clinic:

Please visit Alberta Referral Directory for referral process.

6. Assess Treatment Response

Review the following information to determine your next steps.

- With each C diff episode, it is important to assess the response to anti-CDI therapy. Resolution of diarrhea typically occur within 5-7 days of treatment. C diff recurrence typically happens 2-4 weeks after completing the previous course of anti-CDI therapy.
- Resolution of diarrhea with no recurrence: there is no need to "test for cure" after completing anti-CDI therapy. Repeat testing should only be done if there is recurrence of diarrhea meeting suspected CDI criteria.
- Resolution of diarrhea with recurrence (within 3 months of previous episode): this is considered a CDI recurrence, and the treatment should correspond to the correct episode.
- Partial or no response to treatment: investigate for other causes of diarrhea (chronic diarrhea pathway: www.albertahealthservices.ca/assets/about/scn/ahs-scn-dh-pathway-chronic-diarrhea.pdf).

Advice Options

You can request non-urgent advice at any point when uncertain about medications, next steps in treatment, imaging, or resources available.

Zone	Program	Online Request	Phone Number
**Urgent Telephone**			
All Zones	RAAPID	N/A	**North:**<br>1-800-282-9911 or 780-735-0811<br>**South:**<br>1-800-661-1700 or 403-944-4486
**Non-Urgent Electronic**			
North/ Edmonton/ Central Zone	UAH EDM FMT Program		780-492-8307
Calgary/South Zone	Microbial Therapy Clinic		403-944-6520

Referral Process

Referral pathways are guidelines to help referring providers know what information, labs and diagnostic imaging are required with their referral to a specialty. These pathways are co-designed with Primary and Specialty Care, AHS Operations, and patients to ensure the right amount of information is included throughout the referral process to triage the patient as quickly as possible.

To ensure referring providers have referral information at their fingertips, referral pathways may link to clinical pathways when available. AHS manages referral pathways and extensive work is ongoing as part of the Alberta Surgical Initiative. If you have questions or want to know more about the referral pathway development process, please email access.ereferral@ahs.ca. [4]

- Severe or fulminant cases RAAPID.
- For routine referrals please see section 5

BACKGROUND

About this pathway

- This pathway was developed in collaboration with Primary Care Physicians, AHS Primary Health Care, AHS Public Health and AHS Medical Offices of Health.
- Condition-specific clinical pathways are intended to offer evidence-based guidance to support primary care providers in caring for patients with a range of clinical conditions.
- This pathway includes hyperlinks and is intended to be used as an electronic tool.

Authors and conflict of interest declaration

Names of the content creators and their conflict-of-interest declarations are available on request by emailing albertapathways@primarycarealberta.ca.

Pathway review process, timelines

Primary care pathways undergo scheduled review every three years, or earlier if there is a clinically significant change in knowledge or practice. The next scheduled review is May 2028. However, we welcome feedback at any time. Please send us your feedback here.

DISCLAIMER

This pathway represents evidence-based best practice but does not override the individual responsibility of healthcare professionals to make decisions appropriate to their patients using their own clinical judgment given their patients' specific clinical conditions, in consultation with patients/alternate decision makers. The pathway is not a substitute for clinical judgment or advice of a qualified healthcare professional. It is expected that all users will seek advice of other appropriately qualified and regulated healthcare providers with any issues transcending their specific knowledge, scope of regulated practice or professional competence.

PROVIDER RESOURCES

Chronic Diarrhea Primary Care Pathway	www.albertahealthservices.ca/assets/about/scn/ahs-scn-dh-pathway-chronic-diarrhea.pdf
Bugs and Drugs	www.bugsanddrugs.org/

PATIENT RESOURCES

This section is intended to list resources that primary care providers may find useful to share with patients to help support self-management and care in the medical home.

Fecal transplant for recurrent Clostridioides difficile (C. diff.) infection Video (MyHealth Alberta)	https://myhealth.alberta.ca/health/Pages/HealthVideoPlayer.aspx?List=fde13c02%2D8aa3%2D41ec%2D920d%2Ded3c17022ba8&ID=1437&Web=a1f56890%2D31e7%2D4ead%2Da4e1%2D767df47e7a65
Clostridioides Difficile (C. diff) Colitis (MyHealth Alberta)	https://myhealth.alberta.ca/health/pages/conditions.aspx?Hwid=uf6176spec
Learning About Clostridioides Difficile (C. diff) Infection (MyHealth Alberta)	https://myhealth.alberta.ca/health/AfterCareInformation/pages/conditions.aspx?HwId=abq5222
Clostridioides Difficile (C. diff) Toxins Test (MyHealth Alberta)	https://myhealth.alberta.ca/health/tests-treatments/pages/conditions.aspx?Hwid=abq4854
Learning About a Fecal Transplant (MyHealth Alberta)	https://myhealth.alberta.ca/health/AfterCareInformation/pages/conditions.aspx?HwId=acg4186

© 2025 Primary Care Alberta, Provincial Pathways Unit

This work is licensed under a Creative Commons Attribution-Non-Commercial-Share Alike 4.0 International license. You are free to copy, distribute and adapt the work for non-commercial purposes, as long as you attribute the work to Primary Care Alberta and abide by the other license terms. If you alter, transform, or build upon this work, you may distribute the resulting work only under the same, similar, or compatible license. The license does not apply to content for which the Primary Care Alberta is not the copyright owner.
